# Supplementary material for: Saline aqueous fluid circulation in mantle wedge inferred from olivine wetting properties
Source: Nat Commun. 2019 Dec 5;10:5557. doi: 10.1038/s41467-019-13513-7 (PMC6895192; doi:10.1038/s41467-019-13513-7)
Supplement: Supplementary file 1 — Supplementary Information [file 41467_2019_13513_MOESM1_ESM.pdf]

Supplementary Information for:

Saline aqueous fluid circulation in mantle wedge inferred from olivine wetting properties

by Huang et al.

Supplementary Note 1

Here, we describe detailed procedures of the selection and measurement of the apparent dihedral angles (θ). In each run product, we captured more than 200 images on the polished cross-section by using field emission-type scanning electron microscopes (JEOL JSM7100F and Hitachi High-Technologies S-5500) at accelerating voltages of 15 and 15 keV, respectively. Each image contains only a few apparent dihedral angles because of the high magnification (up to 150,000). We utilized secondary electron (SE) images because they provide information on the three-dimensional morphology of the pores.

For θ measurement, we selected angles in each image that included (1) no cracks or open grain boundaries that formed during the quenching and polishing and (2) a clear cross-point defined by two curved interfaces. To avoid the effect of the faceted crystal plane, we focused on the angle defined by the curved–curved interfaces. In Supplementary Figs. 1 and 2, we show examples of the angle selection.

Measurement for θ was then performed by using Image-J software. We drew two tangent lines along the curved interfaces as shown in Supplementary Fig. 3. For each angle, we repeated the same measurement at least five times and calculated the average. We finally obtained a median of more than 200 measured apparent θ to infer the true θ.

Supplementary Note 2

We briefly discuss the uncertainty of the inferred true θ based on errors in measurement and in the range of data. In each apparent θ, we measured the angle value at least five times and we calculated the average of these values as per measured θ. The discrepancy of five-times measured angle values fell mostly within a range of ±~3°. The median of these apparent dihedral angles could be much smaller owing to the statistical averaging effect. The measurement of more than 200 apparent θ ensured a stable median value. The intrinsic errors associated with the experimental procedures were negligible because the difference in median angle between the two experiments conducted in the same system at the same pressure (P)–temperature (T) conditions in the two laboratories was small (i.e., 56.1° in CDM-04 and 55.8° in CDMR-4 at 1 GPa and 1000 °C).

The median angle coincides with the true θ in the isotropic system with one true θ (Jurewicz and Jurewicz; 1986). In contrast, the median represents the most frequent true θ when the true θ shows a range owing to anisotropy of the crystal surface energy. In our study, we assumed that the dominant true θ has a limited range because the cumulative frequency curves of the apparent θ showed a steep increase around the median angle, which is consistent with the theoretical distribution with one dominant true θ. Therefore, in summary, we expect that the true angle should be within 3° from the median value obtained in this research.

Supplementary Figures

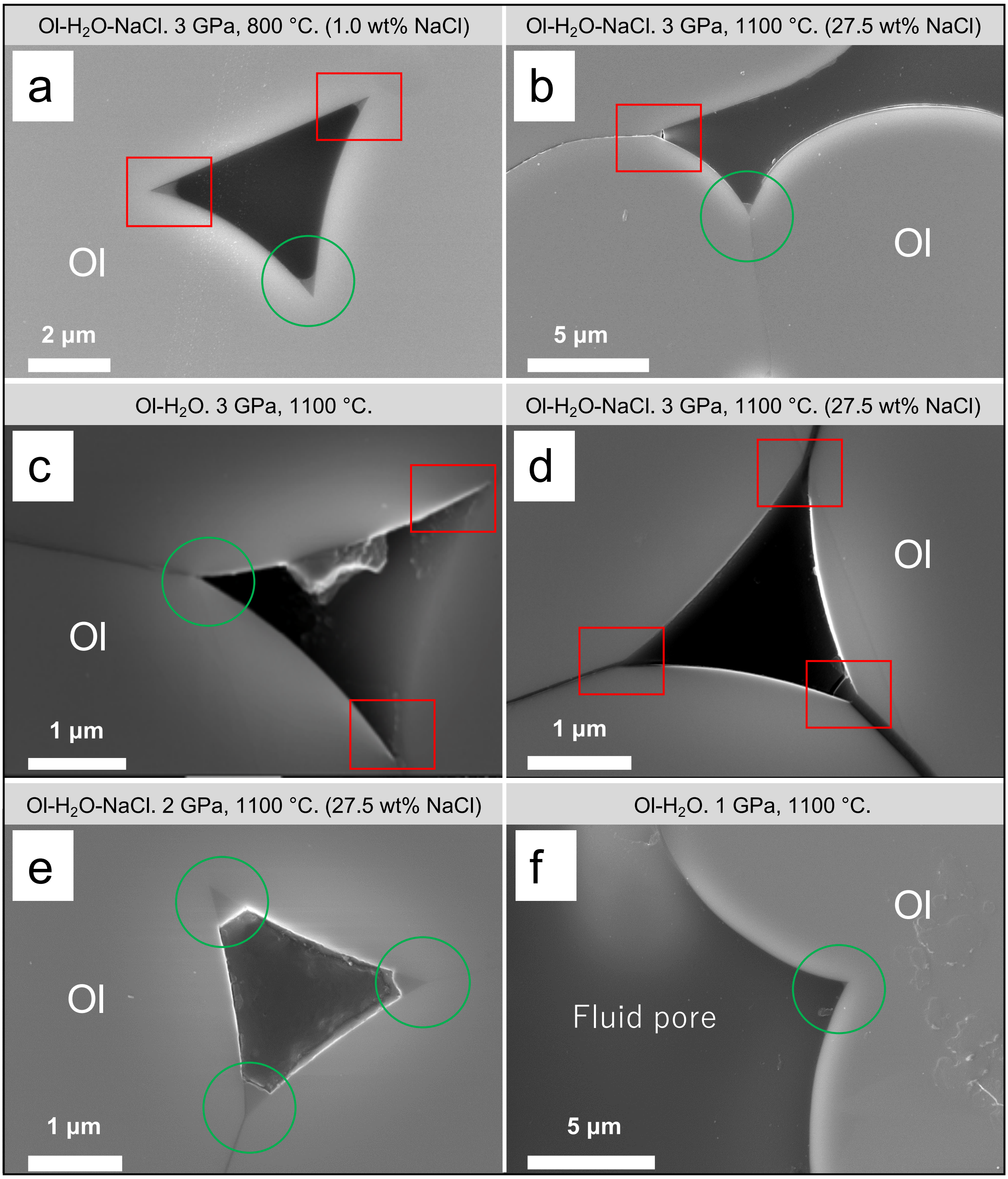

Supplementary Figure 1 | Examples for dihedral angle (θ) measurement. All images are secondary electron images. The gray solid phases are olivine, and the black areas are fluid pores. The experimental condition and system are shown at the top of each image. The θ with

a green circle (red rectangle) represents a suitable (unsuitable) type of angle for measurement because it satisfies (does not satisfy) our requirements.

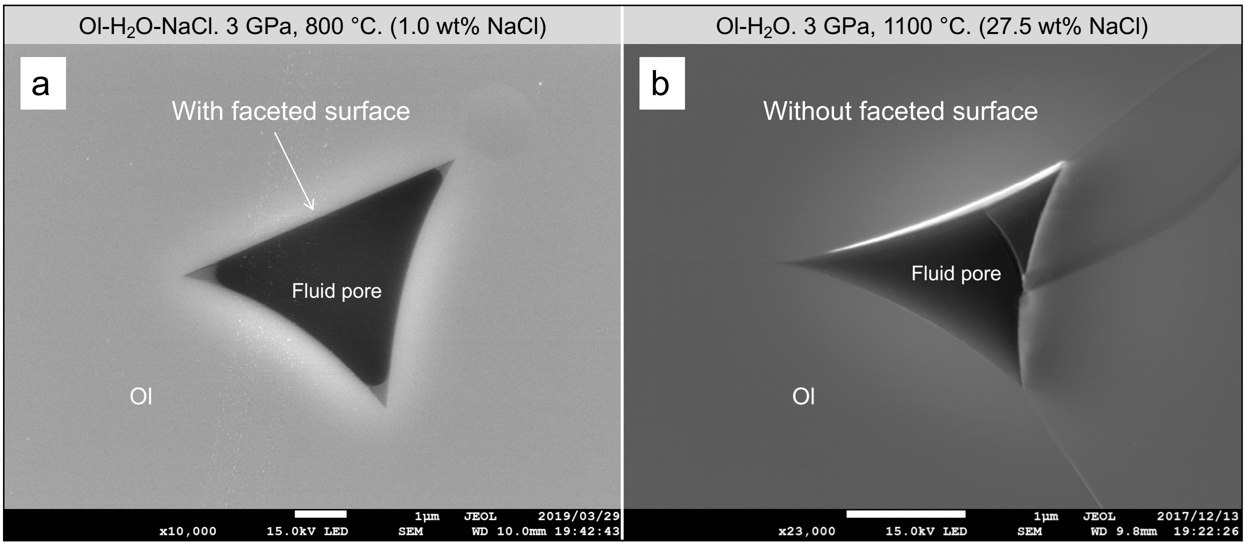

Supplementary Figure 2 | Dihedral angle with and without flat interface. a Secondary electron (SE) images showing a triple junction with one flat interface. **b** SE images showing a triple junction without a flat interface. The gray solid phases are olivine, and the black areas are fluid pores. The experimental condition and system are shown at the top of each image.

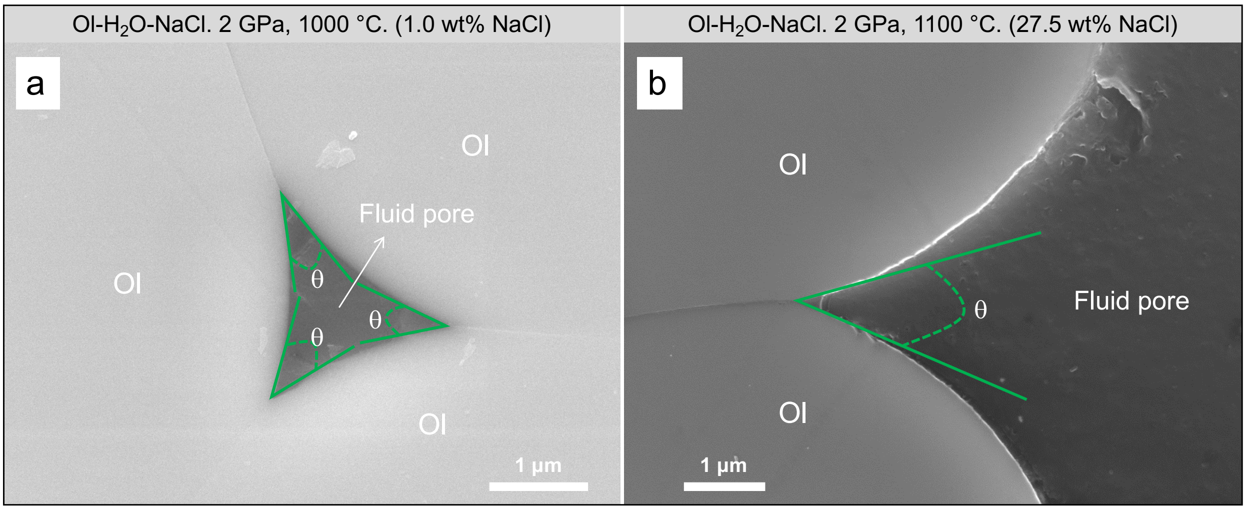

Supplementary Figure 3 | Examples showing method of measurement. a, b Secondary electron (SE) images. The gray solid phases are olivine, and the black areas are fluid pores. The experimental condition and system are shown at the top of each image. The green lines indicate the manner in which we obtained the dihedral angle value from the SE image.

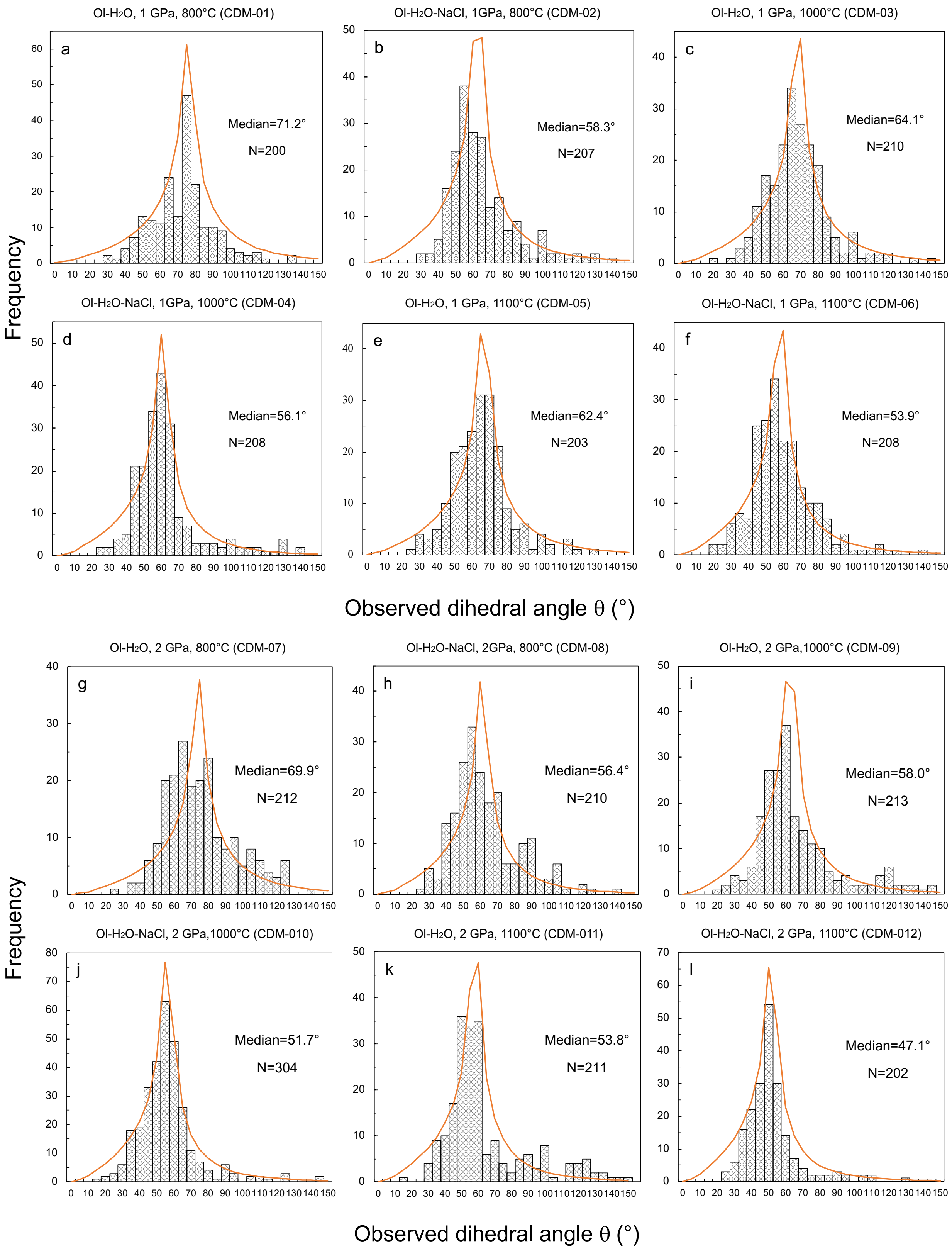
Ol-H2O, 1 GPa, 800°C (CDM-01)
a
Median=71.2°
N=200
Ol-H2O-NaCl, 1GPa, 800°C (CDM-02)
b
Median=58.3°
N=207
Ol-H2O, 1 GPa, 1000°C (CDM-03)
c
Median=64.1°
N=210
Ol-H2O-NaCl, 1GPa, 1000°C (CDM-04)
d
Median=56.1°
N=208
Ol-H2O, 1 GPa, 1100°C (CDM-05)
e
Median=62.4°
N=203
Ol-H2O-NaCl, 1 GPa, 1100°C (CDM-06)
f
Median=53.9°
N=208
Frequency
Observed dihedral angle θ (°)
Ol-H2O, 2 GPa, 800°C (CDM-07)
g
Median=69.9°
N=212
Ol-H2O-NaCl, 2GPa, 800°C (CDM-08)
h
Median=56.4°
N=210
Ol-H2O, 2 GPa,1000°C (CDM-09)
i
Median=58.0°
N=213
Ol-H2O-NaCl, 2 GPa,1000°C (CDM-010)
j
Median=51.7°
N=304
Ol-H2O, 2 GPa, 1100°C (CDM-011)
k
Median=53.8°
N=211
Ol-H2O-NaCl, 2 GPa, 1100°C (CDM-012)
l
Median=47.1°
N=202
Frequency
Observed dihedral angle θ (°)

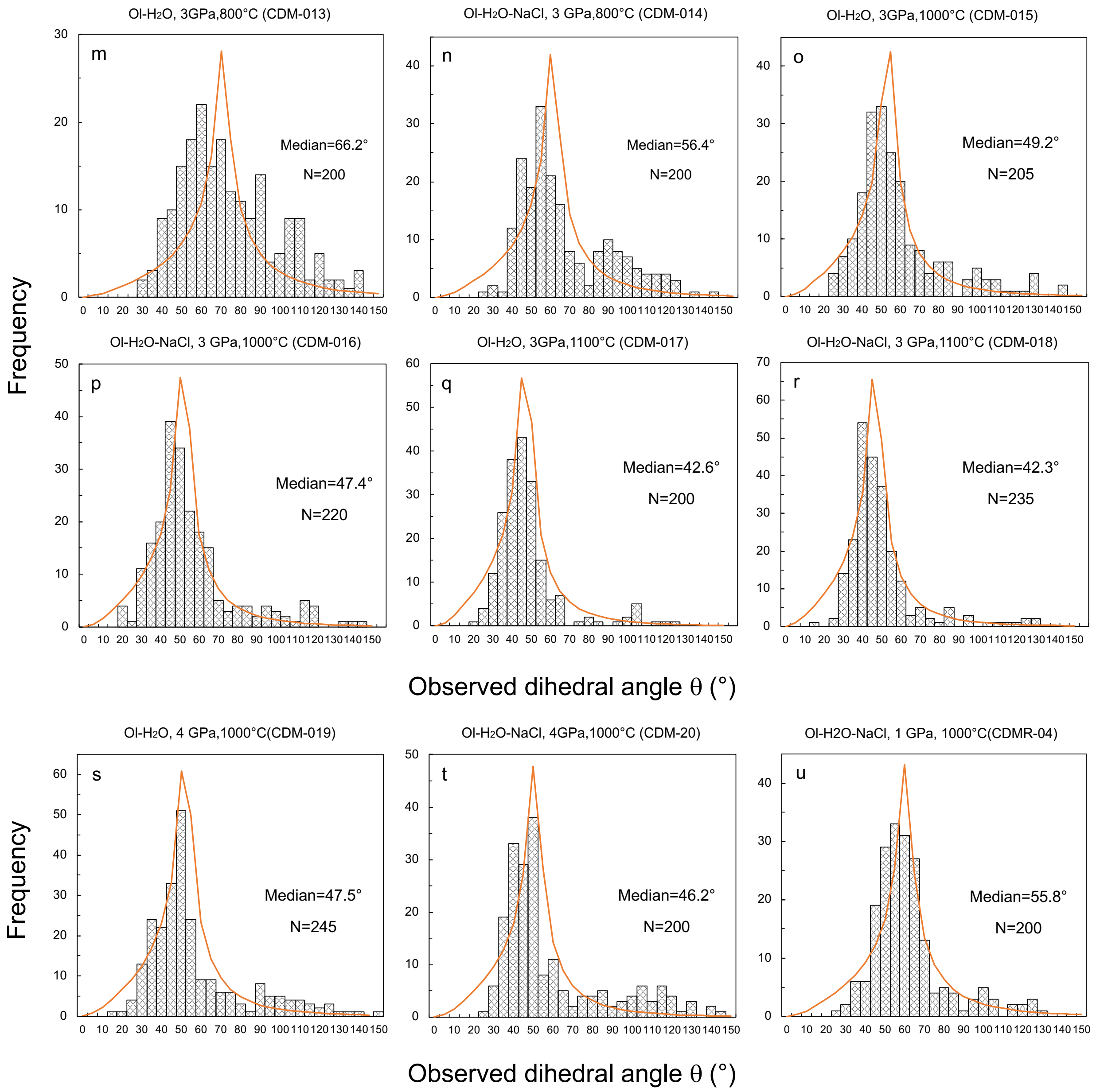

Supplementary Figure 4 | Frequency distribution histograms of measured apparent dihedral angles in olivine–H_2O and olivine–H_2O–NaCl systems (27.5 wt% NaCl) at 1–4 GPa and 800–1100 °C. Theoretical distributions (orange curves) for a mono-mineralic and isotropic system are also shown in the histograms along with the median value (Jurewicz and Jurewicz, 1986).

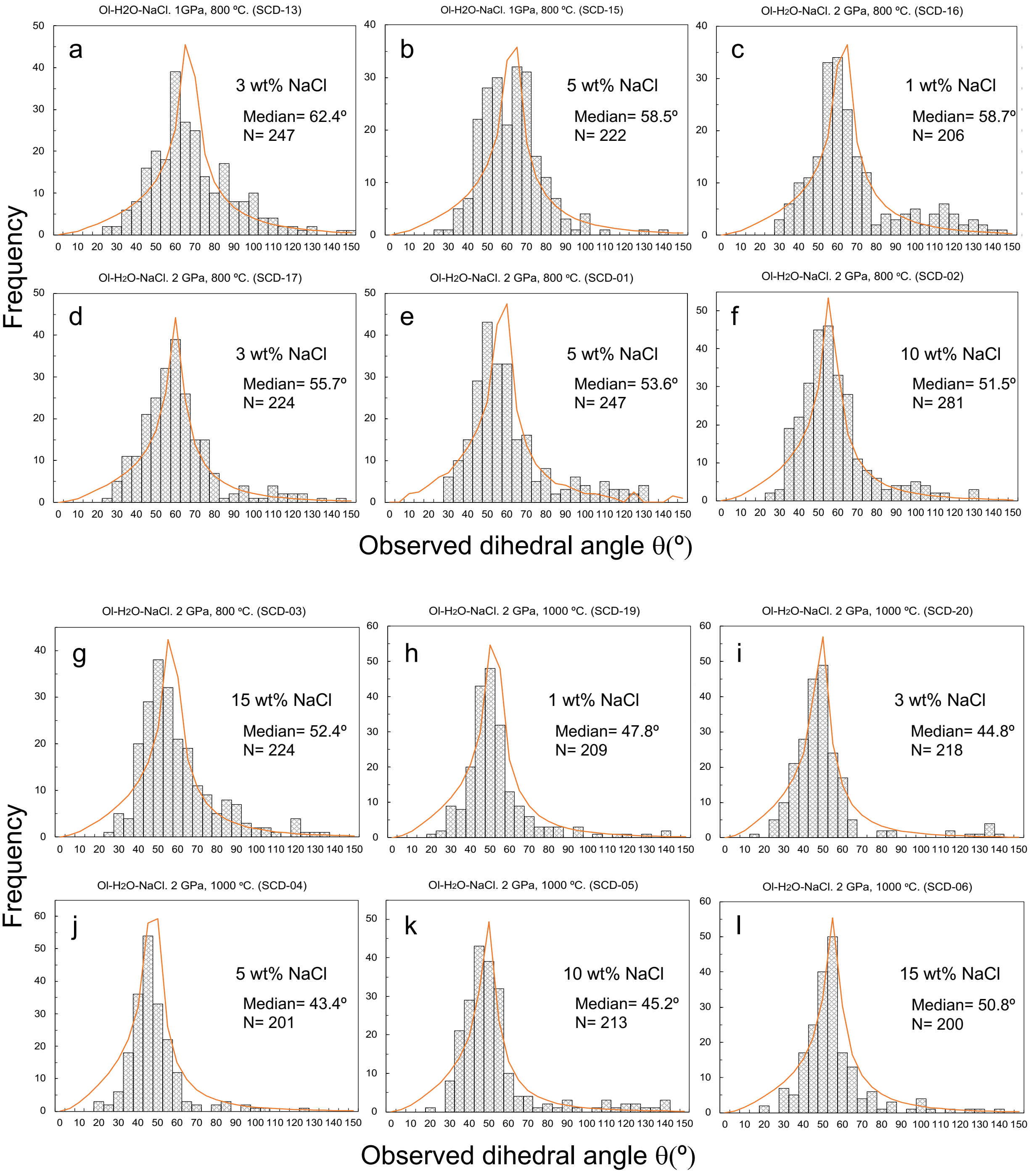
Ol-H2O-NaCl. 1GPa, 800 ºC. (SCD-13)
a
3 wt% NaCl
Median= 62.4º
N= 247
Ol-H2O-NaCl. 1GPa, 800 ºC. (SCD-15)
b
5 wt% NaCl
Median= 58.5º
N= 222
Ol-H2O-NaCl. 2 GPa, 800 ºC. (SCD-16)
c
1 wt% NaCl
Median= 58.7º
N= 206
Ol-H2O-NaCl. 2 GPa, 800 ºC. (SCD-17)
d
3 wt% NaCl
Median= 55.7º
N= 224
Ol-H2O-NaCl. 2 GPa, 800 ºC. (SCD-01)
e
5 wt% NaCl
Median= 53.6º
N= 247
Ol-H2O-NaCl. 2 GPa, 800 ºC. (SCD-02)
f
10 wt% NaCl
Median= 51.5º
N= 281
Frequency
Observed dihedral angle θ(º)
Ol-H2O-NaCl. 2 GPa, 800 ºC. (SCD-03)
g
15 wt% NaCl
Median= 52.4º
N= 224
Ol-H2O-NaCl. 2 GPa, 1000 ºC. (SCD-19)
h
1 wt% NaCl
Median= 47.8º
N= 209
Ol-H2O-NaCl. 2 GPa, 1000 ºC. (SCD-20)
i
3 wt% NaCl
Median= 44.8º
N= 218
Ol-H2O-NaCl. 2 GPa, 1000 ºC. (SCD-04)
j
5 wt% NaCl
Median= 43.4º
N= 201
Ol-H2O-NaCl. 2 GPa, 1000 ºC. (SCD-05)
k
10 wt% NaCl
Median= 45.2º
N= 213
Ol-H2O-NaCl. 2 GPa, 1000 ºC. (SCD-06)
l
15 wt% NaCl
Median= 50.8º
N= 200
Frequency
Observed dihedral angle θ(º)

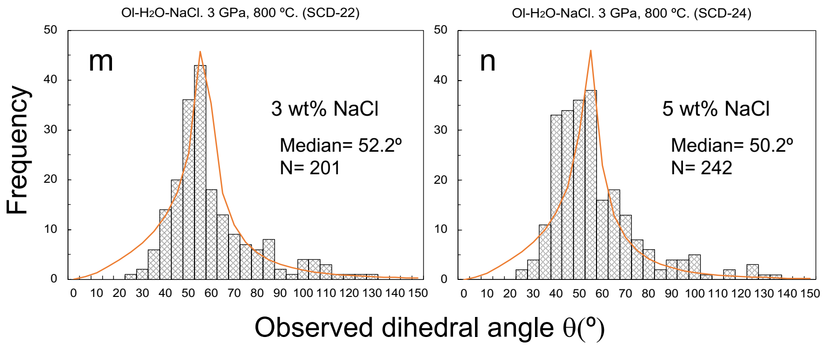

Supplementary Figure 5 | Frequency distribution histograms of measured apparent dihedral angles in olivine–H_2O–NaCl systems with 1.0, 3.0, 5.0, 10.0, and 15.0 wt% NaCl at 800 and 1000 °C under pressures of 1–3 GPa. Theoretical distributions (orange curves) for a mono-mineralic and isotropic system are also shown in the histograms with the median value (Jurewicz and Jurewicz, 1986).

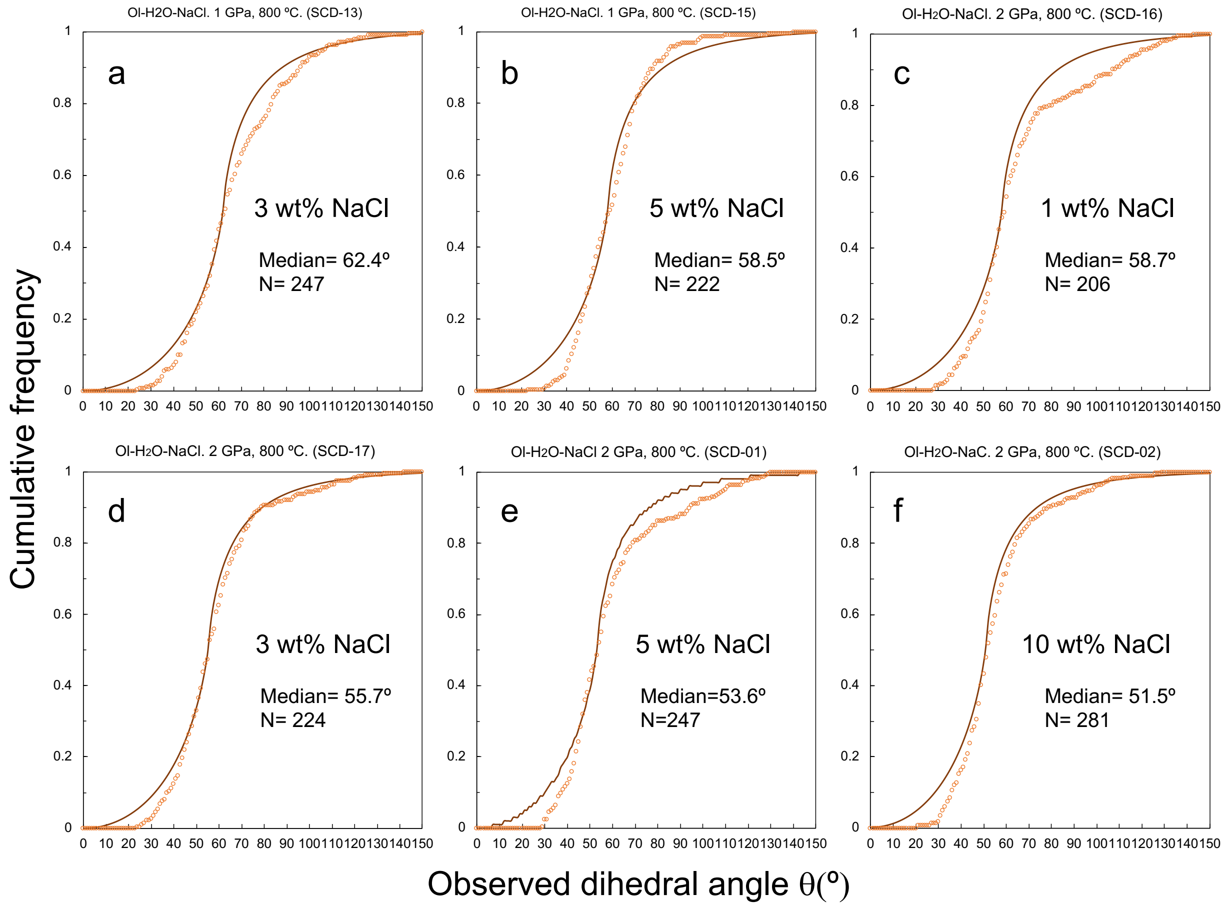

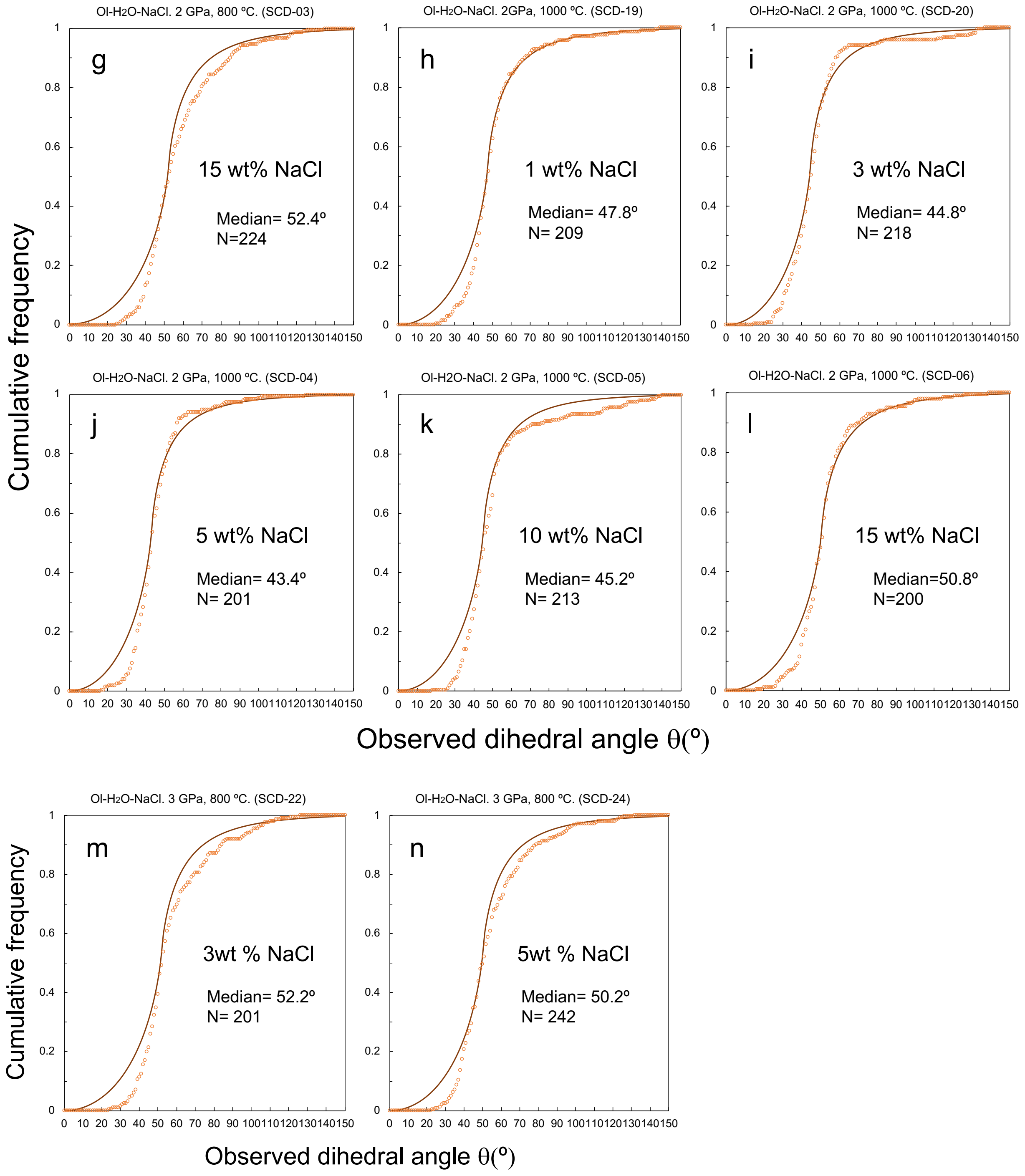

Supplementary Figure 6 | Cumulative frequency curves of measured apparent dihedral angles in the olivine–H_2O–NaCl systems with 1.0, 3.0, 5.0, 10.0, and 15.0 wt% NaCl at 800 and 1000 °C under pressures of 1–3 GPa. Orange circles denote data in the H_2O–NaCl systems with different NaCl concentrations. The median value and number (N) of measured angles are shown for each experimental condition. The thin black lines represent the theoretical cumulative frequency curves of the isotropic system with one true dihedral angle, which is assumed to coincide with the obtained median value (Jurewicz and Jurewicz, 1986).

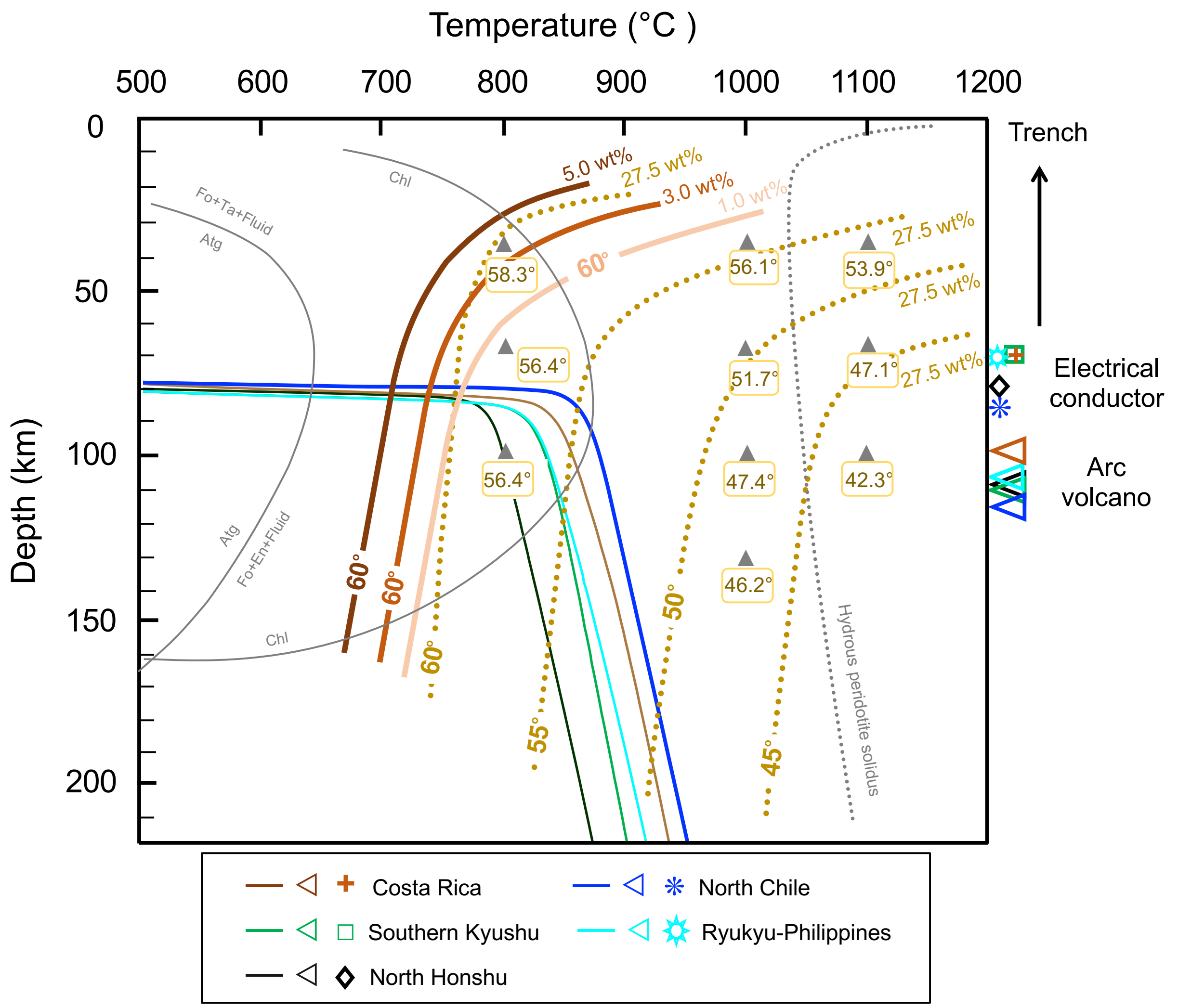

Supplementary Figure 7 | 60° isopleths of the dihedral angle (θ) in olivine–H_2O–NaCl systems with 27.5 wt% NaCl along with the median of measured angles in depth–temperature space. The thick brown, red–orange, and orange curves are the 60° isopleths in the low NaCl concentration systems; the yellowish brown dotted curve is the 60° isopleth for 27.5 wt% NaCl, and the blue dashed curve is the 60° isopleth in the H_2O system. In pressure (P)–temperature (T) regions higher than the 60° isopleths, θ is smaller than 60°. The depth–temperature paths of the down-dragged mantle at the base of the mantle wedge are denoted by five thin solid lines of different colors for intermediate-temperature and cold subduction zones (van Keken et al., 2011; Syracuse et al., 2010), where high electrical conductivity has been detected in the deep fore-arc crust. The slab-surface depths beneath the fore-arc conductor and the arc volcano are shown at the right side of the panel for each subduction zone (England and Katz, 2010). The gray dotted line shows the hydrous peridotite solidus that is after Green (2015). Here it should be noted that this hydrous peridotite solidus was constrained in a fertile

peridotite system; the solidus temperature in a relatively depleted peridotite should be higher than that shown in this figure. The stability of antigorite is after Bromiley and Pawley (2003) and Evans et al. (1976), and the stability of chlorite is after Till et al. (2012). Abbreviations are Atg: antigorite; Fo: forsterite; Ta: talc; En: enstatite; and Chl: chlorite.

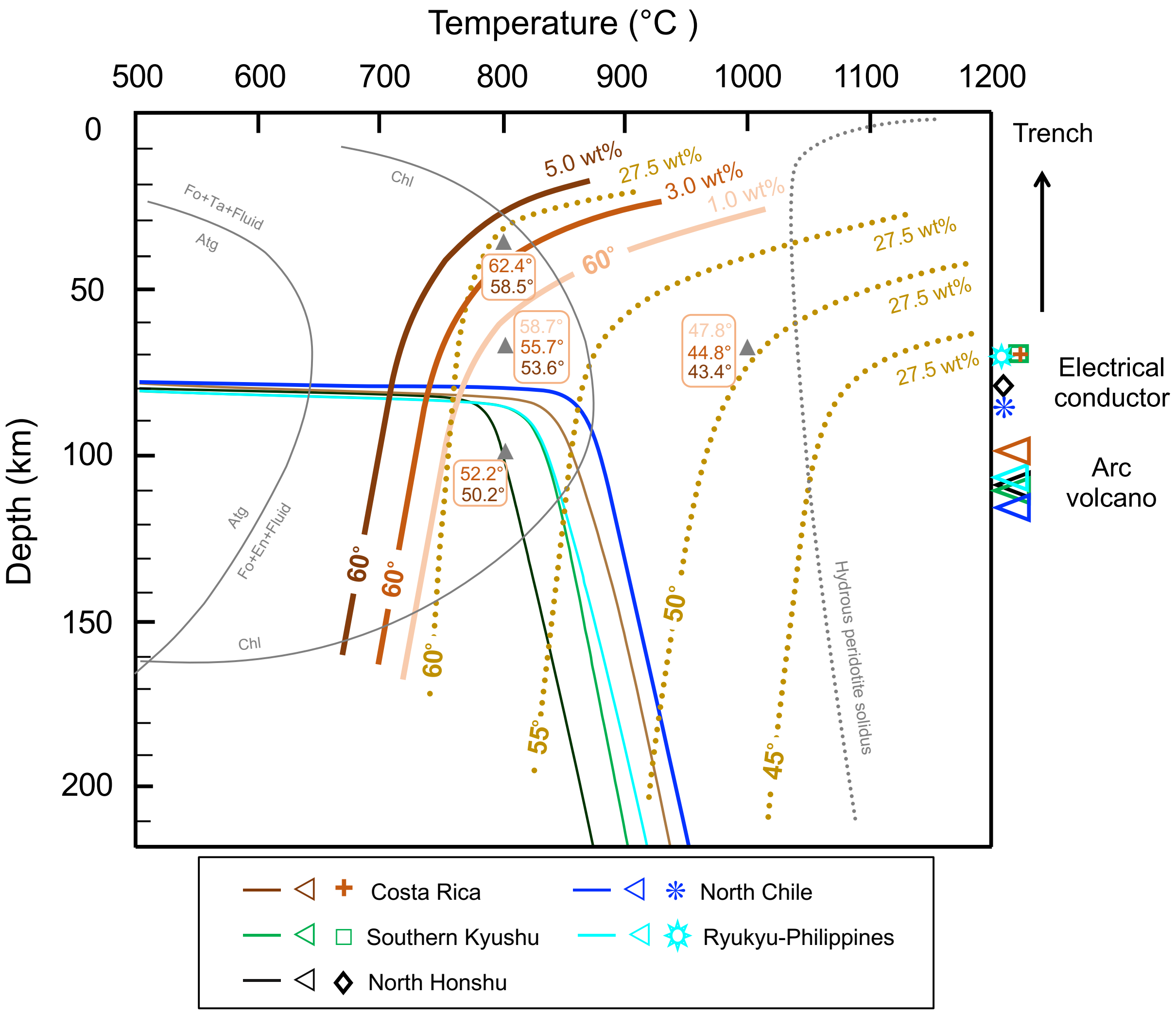

Supplementary Figure 8 | 60° isopleths of the dihedral angles (θ) in olivine–H_2O–NaCl system with 1.0, 3.0, and 5.0 wt% NaCl along with the median of the measured angles in depth–temperature space. Although we lack the data for 1 wt% NaCl at 800 °C and 1.0 and 3.0 GPa, we made two assumptions to roughly estimate the 60° isopleth for 1 wt% NaCl according to the pressure (P)–temperature (T)–NaCl concentration dependence in Fig. 4: (1) the median angles at 1.0 and 3.0 GPa are larger and smaller, respectively, than that at 2.0 GPa; (2) the median angle value with 1 wt% NaCl fell between those with no NaCl and 3.0 wt% NaCl. The thick brown, red–orange, and orange curves are the 60° isopleths in the low NaCl concentration systems; the yellowish brown dotted curve is the 60° isopleth for the 27.5 wt%

NaCl, and the blue dashed curve is the 60° isopleth in the H_2O system. In P–T regions higher than the 60° isopleths, the dihedral angle is smaller than 60°. The depth–temperature paths of down-dragged mantle at the base of mantle wedge is denoted by five thin solid lines of different colors for intermediate-temperature and cold subduction zones (van Keken et al., 2011; Syracuse et al., 2010), where high electrical conductivity has been detected in the deep fore-arc crust. The slab-surface depths beneath the fore-arc conductor and the arc volcano are shown at the right side of the panel for each subduction zone (England and Katz, 2010). The gray dotted line shows the hydrous peridotite solidus that is after Green (2015). Here, it should be noted that this hydrous peridotite solidus was constrained in a fertile peridotite system; the solidus temperature in a relatively depleted peridotite should be higher than that shown in this figure. The stability of antigorite is after Bromiley and Pawley (2003) and Evans et al. (1976), and the stability of chlorite is after Till et al. (2012). Abbreviations are Atg: antigorite; Fo: forsterite; Ta: talc; En: enstatite; and Chl: chlorite.

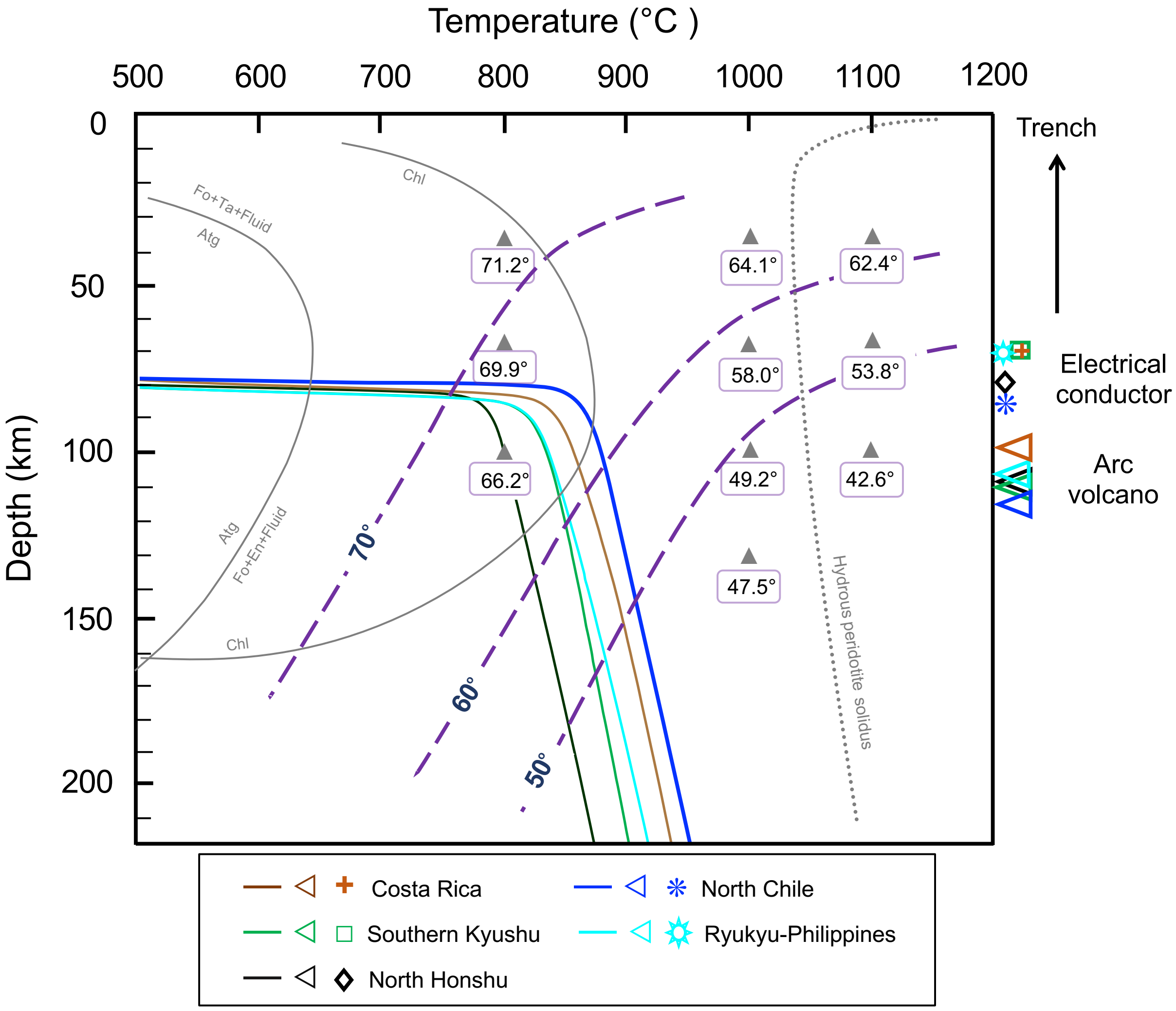

Supplementary Figure 9 | Isopleths of the dihedral angles (θ) in the olivine–H_2O system along with the median of measured angles in depth–temperature space. The purple dashed curves are the 60° isopleths in the H_2O systems. The depth–temperature paths of down-dragged mantle at the base of mantle wedge are denoted by five thin solid lines of different colors for intermediate-temperature and cold subduction zones (van Keken et al., 2011; Syracuse et al., 2010), where high electrical conductivity has been detected in the deep fore-arc crust. The slab-surface depths beneath the fore-arc conductor and the arc volcano are shown at the right side of the panel for each subduction zone (England and Katz, 2010). The gray dotted line shows the hydrous peridotite solidus that is after Green (2015). Here, it should be noted that this hydrous peridotite solidus was constrained in a fertile peridotite system; the solidus temperature in a relatively depleted peridotite should be higher than that shown in this figure. The stability of antigorite is after Bromiley and Pawley (2003) and Evans et al. (1976), and the stability of chlorite is after Till et al. (2012). Abbreviations are Atg: antigorite; Fo: forsterite; Ta: talc; En: enstatite; and Chl: chlorite.

Supplementary Table

Supplementary Table 1 | Experimental conditions and results.

Run No.	P (GPa)	T (°C)	Duration (h)	System	Salinity (wt%)	Number of angles measured	Median (°.)
CDM-01	1	800	192	H_2O	0	200	71.2
CDM-03	1	1000	120	H_2O	0	210	64.1
CDM-05	1	1100	72	H_2O	0	203	62.4
CDM-07	2	800	210	H_2O	0	212	69.9
CDM-09	2	1000	120	H_2O	0	213	58.0
CDM-11	2	1100	72	H_2O	0	211	53.8
CDM-13	3	800	211	H_2O	0	200	66.2
CDM-15	3	1000	120	H_2O	0	205	49.2
CDM-17	3	1100	72	H_2O	0	200	42.6
CDM-19	4	1000	120	H_2O	0	245	47.5
CDM-02	1	800	192	NaCl aq.	27.5	207	58.3
CDM-04	1	1000	120	NaCl aq.	27.5	208	56.1
CDMR-4	1	1000	120	NaCl aq.	27.5	200	55.8
CDM-06	1	1100	72	NaCl aq.	27.5	208	53.9
CDM-08	2	800	210	NaCl aq.	27.5	210	56.4
CDM-10	2	1000	120	NaCl aq.	27.5	304	51.7
CDM-12	2	1100	72	NaCl aq.	27.5	202	47.1
CDM-14	3	800	211	NaCl aq.	27.5	200	56.4
CDM-16	3	1000	120	NaCl aq.	27.5	220	47.4
CDM-18	3	1100	72	NaCl aq.	27.5	235	42.3
CDM-20	4	1000	120	NaCl aq.	27.5	200	46.2
SCD-13	1	800	192	NaCl aq.	3.0	247	62.4
SCD-15	1	800	192	NaCl aq.	5.0	222	58.5
SCD-16	2	800	192	NaCl aq.	1.0	206	58.7
SCD-17	2	800	192	NaCl aq.	3.0	224	55.7
SCD-01	2	800	192	NaCl aq.	5.0	247	53.6
SCD-02	2	800	192	NaCl aq.	10.0	281	51.5
SCD-03	2	800	192	NaCl aq.	15.0	224	52.4
SCD-19	2	1000	140	NaCl aq.	1.0	209	47.8
SCD-20	2	1000	140	NaCl aq.	3.0	218	44.8
SCD-04	2	1000	133	NaCl aq.	5.0	201	43.4
SCD-05	2	1000	133	NaCl aq.	10.0	213	45.2
SCD-06	2	1000	133	NaCl aq.	15.0	200	50.8
SCD-22	3	800	192	NaCl aq.	3.0	201	52.2
SCD-24	3	800	192	NaCl aq.	5.0	242	50.2

NaCl aq.: NaCl aqueous solution with different concentrations.

Reference

1. Jurewicz, S. R., Jurewicz, A. J. Distribution of apparent angles on random sections with emphasis on dihedral angle measurements. *J. Geophys. Res.* **91**, 9277–9282 (1986).
2. van Keken, P. E., Hacker, B. R., Syracuse, E. M., Abers, G. A. Subduction factory: 4. Depth-dependent flux of H_2O from subducting slabs worldwide. *J. Geophys. Res.* **116**, (B1) (2011).
3. Syracuse, E. M., van Keken, P. E., Abers, G. A. The global range of subduction zone thermal models. *Phys. Earth Planet. Inter.* **183**, 73–90 (2010).
4. England, P. C., Katz, R. F. Melting above the anhydrous solidus controls the location of volcanic arcs. *Nature.* **467**, 700 (2010).
5. Green, D. H. Experimental petrology of peridotites, including effects of water and carbon on melting in the Earth's upper mantle. *Phys. Chem. Miner*. **42** (2), 95-122 (2015).
6. Till, C. B., Grove, T. L., Withers, A. C. The beginnings of hydrous mantle wedge melting. *Contrib. Mineral. Petrol*. **163** (4), 669–688 (2012).
7. Bromiley, G. D., Pawley, A. R. The stability of antigorite in the systems MgO-SiO_2-H_2O (MSH) and MgO-Al_2O_3-SiO_2-H_2O (MASH): The effects of Al^{3+} substitution on high-pressure stability. *Am. Mineral*. **88** (1), 99-108 (2003).
8. Evans, B. W. Stability of chrysotile and antigorite in the serpentine multisystem. *Schweiz Mineral Petrogr. Mitt*. **56**, 79–93 (1976).
